# Comparative analysis of cervical cancer burden trends between China and G20 countries from 1990 to 2023 with projections to 2035: A population-based observational study

**DOI:** 10.1097/MD.0000000000048640

**Published:** 2026-05-08

**Authors:** Jing Li, Ying Wang, Zhi Xu, Sisi Ao

**Affiliations:** a Department of Obstetrics and Gynecology, Zhangjiagang Fifth People’s Hospital, Zhangjiagang, Jiangsu, China; b Center for Reproductive Medicine, The First Affiliated Hospital of Anhui Medical University, Hefei, Anhui, China; c Science and Education Department, Zhangjiagang Fifth People’s Hospital, Zhangjiagang, Jiangsu, China.

**Keywords:** cervical cancer, disease burden, G20 countries, projection, trend analysis

## Abstract

This study aimed to systematically compare and analyze the trends in cervical cancer disease burden between China and G20 countries from 1990 to 2023 based on the Global Burden of Disease (GBD) 2023 database and project the trajectory through 2035, thereby providing scientific evidence for optimizing cervical cancer prevention and control strategies. Data were sourced from the GBD 2023 database. Age-standardized incidence rate, mortality rate, and disability-adjusted life years (DALYs) rate of cervical cancer among females in China and G20 countries were selected as study indicators. The estimated annual percentage change (EAPC) was used to assess temporal trends in disease burden; decomposition analysis was employed to quantify the contributions of population growth, aging, and epidemiological changes; frontier analysis was conducted based on the socio-demographic index (SDI); and a Bayesian age-period-cohort model was applied to project mortality in 2035. From 1990 to 2023, the age-standardized incidence rate of cervical cancer in China declined from 17.32 per 100,000 to 12.06 per 100,000 (EAPC: −1.04), with a 52.4% reduction in mortality rate, significantly outperforming the 31.8% reduction observed in G20 countries. China’s DALYs rate decreased from 294.14 per 100,000 to 133.06 per 100,000 (EAPC: −2.43). Substantial heterogeneity was observed among G20 member states, with South Africa recording the highest incidence rate (47.54 per 100,000) and Italy exhibiting an anomalous increase in mortality. Both China and G20 countries showed peak cervical cancer burden in the 50 to 54 age group. Decomposition analysis revealed that epidemiological improvements in China (−128.58%) successfully offset the combined effects of aging (+91.35%) and population growth (+137.22%), whereas G20 countries achieved only partial counterbalancing. Frontier analysis indicated that China’s actual DALYs rate approached the theoretical optimum corresponding to its SDI level. Projections suggest that cervical cancer deaths in China will decline by 40.9% from 2023 to 2035, compared to only 7.9% in G20 countries. China has achieved remarkable progress in cervical cancer prevention and control, with epidemiological improvements effectively offsetting the adverse impacts of demographic factors. Nevertheless, continued efforts to strengthen human papillomavirus vaccination and screening coverage remain essential to achieve the strategic goal of cervical cancer elimination.

## 1. Introduction

Cervical cancer is the fourth most common malignant tumor among women worldwide and one of the leading causes of cancer-related mortality in females.^[1]^ The disease is primarily caused by persistent infection with human papillomavirus (HPV), with high-risk HPV types – particularly HPV16 and HPV18 – associated with approximately 70% of cervical cancer cases.^[2]^ Although cervical cancer is currently the only malignancy with a clearly established etiology that can be effectively prevented through vaccination and screening, it continues to impose a substantial disease burden globally. According to 2022 global cancer statistics, approximately 662,000 new cases of cervical cancer are diagnosed and approximately 348,000 deaths occur annually worldwide, with over 85% of cases occurring in low- and middle-income countries.^[3,4]^

In China, cervical cancer represents a major public health threat to women’s health. In recent years, driven by accelerating urbanization, changing lifestyles, and rising HPV infection rates, cervical cancer in China has shown a trend toward younger age at onset.^[5]^ To address this challenge, the Chinese government launched a free screening program for “two cancers” (cervical cancer and breast cancer) in rural areas in 2009 and piloted the inclusion of HPV vaccination in the national immunization program in 2019, achieving notable results.^[6,7]^ Nevertheless, cervical cancer prevention and control in China continues to face considerable challenges due to uneven regional economic development, disparities in healthcare resource allocation, and variable levels of public health awareness. The incidence and mortality of cervical cancer are influenced by multiple factors, including HPV vaccination coverage, the reach of screening programs, accessibility of healthcare services, and socioeconomic development.^[8]^ In 2020, the World Health Organization (WHO) released the Global Strategy to Accelerate the Elimination of Cervical Cancer, setting targets to achieve, by 2030, 90% of girls vaccinated against HPV before age 15, 70% of women screened with a high-performance test between ages 35 and 45, and 90% of women with cervical disease receiving treatment.^[9]^ While this strategy has charted a clear course for global cervical cancer prevention and control, progress toward these targets varies markedly across countries.

As the world’s major economies, G20 countries encompass nations at varying levels of development, with diverse health systems and cultural contexts. Their experiences in cervical cancer prevention and control, along with trends in disease burden, hold important reference value for the formulation of global public health policies.^[10]^ Systematic comparative studies on cervical cancer burden between China and G20 countries remain relatively scarce, and dynamic comparative analyses of long-term trends in disease burden, demographic drivers, and future projections are particularly insufficient.^[10]^ Accordingly, this study draws on the Global Burden of Disease (GBD) 2023 database to systematically compare and analyze trends in cervical cancer disease burden between China and G20 countries from 1990 to 2023, with a focus on assessing the temporal patterns and age distribution of key indicators including incidence rate, mortality rate, and disability-adjusted life years (DALYs). The findings are intended to provide a scientific basis for developing more targeted cervical cancer prevention and control strategies in China, while offering important epidemiological support for evaluating the feasibility of the cervical cancer elimination goal and optimizing the allocation of public health resources.

## 2. Materials and methods

### 2.1. Data sources

The data for this study were obtained from the GBD 2023 database, published by the Institute for Health Metrics and Evaluation at the University of Washington. This database encompasses incidence, mortality, and disease burden indicators for a wide range of conditions across 204 countries and territories worldwide from 1990 to 2023. China and the G20 countries were designated as the primary study populations, with cervical cancer as the disease of interest. Based on data spanning 1990 to 2023, this study adopted the incidence rate, mortality rate, and DALYs of cervical cancer among females of all ages in China and G20 countries as indicators of disease burden. Age-standardized rates and their corresponding 95% uncertainty intervals were used for all indicators to eliminate the influence of variable age distributions across populations and time periods, thereby ensuring comparability.^[11]^ This study constitutes a secondary analysis of publicly available GBD data, involves no original human subjects research, and complies with the ethical requirements of the Declaration of Helsinki and its subsequent revisions.

### 2.2. Disease definition

Under the International Classification of Diseases, Tenth Revision, cervical cancer is coded as C53 (malignant neoplasm of the cervix uteri).^[12]^ The GBD 2023 study systematically assessed cervical cancer based on this diagnostic code to ensure accuracy and consistency in disease definition.

### 2.3. Statistical methods

All statistical analyses and visualizations were performed using R software (version 4.4.2), with a significance level set at *P* < .05. Figures were generated using the ggplot2 package. All rates were calculated using age-standardization methods with the World Standard Population as the reference population, to eliminate the influence of differences in population age structure.

#### 2.3.1. Trend analysis

The estimated annual percentage change (EAPC) and its 95% confidence intervals provided by the GBD 2023 database were used to assess the long-term trends in cervical cancer disease burden indicators – including age-standardized incidence rate, age-standardized mortality rate (ASMR), and age-standardized DALYs rate – in China and G20 countries from 1990 to 2023. The EAPC was calculated based on a log-linear regression model using the formula EAPC = (e^β−1^) × 100%, where β is the slope of the regression coefficient in the model ln(Y) ~ Year. An EAPC > 0 indicates an upward trend, while an EAPC < 0 indicates a downward trend.^[13]^

#### 2.3.2. Decomposition analysis

Decomposition analysis was applied to attribute changes in cervical cancer case counts and DALYs to 3 key factors: population growth, population aging, and epidemiological change. This approach is based on the Das Gupta decomposition technique and quantifies the contribution of each factor using the following formula: total change = population size effect + age structure effect + age-specific incidence (or mortality) rate effect. Population growth reflects the impact of changes in total population size; population aging captures the effect of shifts in age structure; and epidemiological change, after adjustment for population size and age, reflects the influence of medical interventions and preventive measures on disease burden. The analysis quantified the absolute and relative contributions of each factor to changes in case counts and DALYs, with results expressed in both absolute numbers and percentages. The contribution was calculated as: contribution (%) = (change attributable to a given factor/ total change) × 100%. Positive values indicate that a factor contributed to an increase in disease burden, while negative values indicate that a factor contributed to a reduction in disease burden.^[14]^

#### 2.3.3. Frontier analysis

To evaluate the alignment between each country’s cervical cancer prevention and control performance and its level of socioeconomic development, frontier analysis was employed. This method constructs a health efficiency frontier curve based on the relationship between the socio-demographic index (SDI) and the age-standardized DALYs rate. The frontier line marks the lowest (optimal) age-standardized DALYs rate achievable at a given SDI level. The effective distance of each country from the frontier quantifies the gap between its actual performance and the theoretical optimum. In addition, Spearman correlation analysis was used to evaluate the associations between SDI and both the DALYs rate and the EAPC.^[15]^

#### 2.3.4. Disease burden projection

A Bayesian age-period-cohort (BAPC) model was applied to project cervical cancer mortality rates and death counts through 2035. This model employs Integrated Nested Laplace Approximation for Bayesian inference, enabling effective integration of age effects, period effects, and cohort effects to capture the complex patterns of disease burden variation across age, time, and generation. Mortality was modeled using a Poisson likelihood function, and uncertainty intervals for the projections were quantified. Results are presented as point estimates with their corresponding 95% confidence intervals.^[16]^

## 3. Results

### 3.1. Overall overview of cervical cancer burden in China and G20 countries

**Figure 1.**
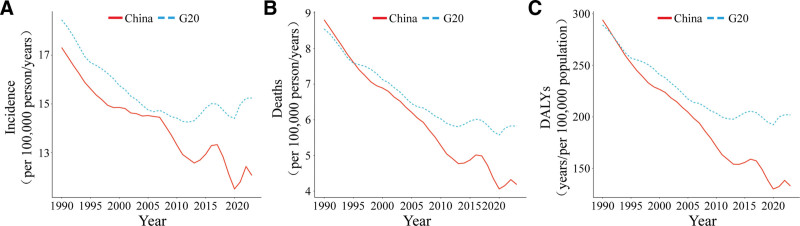
Trends in age-standardized incidence rates of cervical cancer in China and G20 countries from 1990 to 2023.

From 1990 to 2023, the age-standardized incidence rate of cervical cancer in China declined from 17.32 (95% uncertainty interval [UI]: 12.51–25.01) per 100,000 to 12.06 (95% UI: 7.44–15.96) per 100,000 (EAPC: −1.04, 95% confidence interval [CI]: −1.14–−0.94). By comparison, G20 countries experienced a slower decline, from 18.45 (95% UI: 14.86–22.74) per 100,000 to 15.24 (95% UI: 12.25–19.66) per 100,000 (Fig. 1A). The ASMR in China fell from 8.80 (95% UI: 6.45–12.75) per 100,000 to 4.19 (95% UI: 2.67–5.48) per 100,000, representing a reduction of 52.4%, substantially exceeding the 31.8% reduction observed in G20 countries over the same period (Fig. 1B). China’s age-standardized DALYs rate decreased from 294.14 (95% UI: 215.88–420.49) per 100,000 to 133.06 (95% UI: 83.97–173.65) per 100,000 (EAPC: −2.43, 95% CI: −2.54–−2.32), surpassing the G20 average (Fig. 1C). Substantial heterogeneity was observed among G20 member states. South Africa recorded the highest incidence rate in 2023 [47.54 (95% UI: 34.20–69.67) per 100,000] with an EAPC of 3.19 (95% CI: 2.75–3.62), while Mexico achieved the greatest reduction [EAPC: −3.02 (95% CI: −3.25–−2.80)]. Regarding mortality, the Republic of Korea had the most favorable EAPC [−2.92 (95% CI: −3.11–−2.73)], whereas Italy showed an anomalous upward trend [EAPC: 2.06 (95% CI: 1.40–2.73)] (Table 1).

**Table 1:** Trends in cervical cancer incidence rate, mortality rate, and disability-adjusted life years (DALYs) among G20 member states in 1990 and 2023, with estimated annual percentage change (EAPC, 1990–2023) (95% uncertainty interval, per 100,000 population).

Member state	Incidence			Death rate			DALYs		
	1990	2023	EAPC	1990	2023	EAPC	1990	2023	EAPC
Argentina	24.18 (20.56, 28.70)	28.99 (23.78, 34.33)	0.41 (0.21, 0.61)	10.75 (9.47, 12.24)	10.00 (8.51, 11.49)	−0.32 (−0.52, −0.11)	383.64 (338.61, 435.43)	362.63 (308.34, 411.30)	−0.24 (−0.43, −0.05)
Australia	12.77 (11.15, 14.83)	9.66 (7.92, 11.37)	−0.51 (−0.79, −0.22)	3.81 (3.38, 4.24)	2.03 (1.74, 2.32)	−1.76 (−2.02, −1.51)	129.21 (114.85, 143.92)	67.61 (58.56, 76.67)	−1.73 (−2.04, −1.42)
Brazil	23.82 (20.73, 27.84)	19.79 (17.17, 22.82)	−0.89 (−1.07, −0.71)	12.94 (11.42, 14.57)	7.78 (7.21, 8.41)	−1.75 (−1.86, −1.65)	414.43 (366.39, 465.42)	271.07 (254.53, 289.43)	−1.56 (−1.71, −1.41)
Canada	15.03 (12.24, 18.23)	14.71 (11.62, 18.28)	−0.15(−0.33, 0.03)	3.64 (3.25, 4.06)	2.49 (2.17, 2.79)	−1.28 (−1.44, −1.13)	118.76 (105.62, 133.06)	87.30 (75.21, 98.36)	−1.04 (−1.23, −0.86)
China	17.32 (12.51, 25.01)	12.06 (7.44, 15.96)	−1.04 (−1.14, −0.94)	8.80 (6.45, 12.75)	4.19 (2.67, 5.48)	−2.27 (−2.39, −2.16)	294.14 (215.88, 420.49)	133.06 (83.97, 173.65)	−2.43 (−2.54, −2.32)
EU	13.99 (12.52, 15.83)	9.94 (8.49, 11.24)	−1.03 (−1.08, −0.98)	6.09 (5.66, 6.57)	3.18 (2.89, 3.45)	−1.93 (−1.98, −1.88)	196.19 (184.93, 209.37)	101.18 (91.22, 109.77)	−2.02 (−2.06, −1.98)
France	11.65 (9.51, 13.53)	9.32 (7.48, 11.35)	−0.74 (−0.84, −0.64)	4.78 (4.17, 5.50)	2.67 (2.28, 3.05)	−1.77 (−1.87, −1.66)	145.83 (130.11, 166.98)	82.84 (71.35, 93.82)	−1.75 (−1.86, −1.64)
Germany	14.67 (12.70, 17.11)	9.53 (8.36, 10.91)	−1.40 (−1.76, −1.04)	6.28 (5.70, 6.95)	2.91 (2.59, 3.24)	−2.45 (−2.84, −2.06)	192.58 (177.53, 210.24)	95.18 (86.62, 106.11)	−2.20 (−2.60, −1.80)
India	23.23 (13.47, 34.32)	19.33 (12.81, 29.45)	−0.74 (−0.99, −0.49)	14.86 (8.82, 21.55)	10.07 (6.68, 15.42)	−1.34 (−1.54, −1.14)	499.95 (291.15, 721.71)	333.17 (226.59, 508.69)	−1.41 (−1.63, −1.18)
Indonesia	10.85 (6.50, 17.51)	16.09 (9.52, 26.83)	1.51 (1.27, 1.74)	5.93 (3.54, 9.81)	6.96 (4.12, 11.10)	0.80 (0.59, 1.01)	215.75 (129.72, 351.68)	267.31 (158.53, 427.06)	1.00 (0.79, 1.22)
Italy	4.86 (3.96, 5.93)	8.94 (6.87, 10.99)	2.99 (2.32, 3.66)	1.69 (1.45, 1.96)	2.21 (1.87, 2.56)	2.06 (1.40, 2.73)	53.44 (45.63, 61.63)	69.90 (59.41, 81.11)	1.98 (1.38, 2.58)
Japan	11.05 (9.46, 13.12)	13.29 (11.16, 15.38)	0.80 (0.67, 0.93)	3.75 (3.25, 4.30)	3.00 (2.56, 3.37)	−0.71 (−0.86, −0.56)	110.56 (97.20, 125.09)	106.01 (91.90, 118.73)	−0.09 (−0.18, 0.00)
Mexico	40.36 (34.88, 46.97)	16.45 (14.04, 18.85)	−3.02 (−3.25, −2.80)	23.24 (20.69, 26.26)	7.04 (6.25, 7.83)	−3.86 (−4.04, −3.69)	706.35 (632.30, 794.52)	230.57 (204.28, 254.22)	−3.59 (−3.81, −3.37)
South Korea	13.26 (9.03, 17.71)	9.57 (6.70, 15.88)	−1.14 (−1.24, −1.04)	5.77 (4.18, 8.13)	2.52 (1.90, 4.12)	−2.92 (−3.11, −2.73)	194.81 (134.95, 260.90)	78.75 (57.91, 128.84)	−3.03 (−3.16, −2.90)
Russia	12.85 (10.62, 15.13)	17.29 (14.31, 20.81)	1.33 (1.10, 1.56)	6.54 (5.69, 7.52)	5.40 (4.72, 6.13)	−0.42 (−0.57, −0.26)	202.01 (175.64, 232.89)	202.10 (177.60, 231.11)	0.21 (−0.02, 0.43)
Saudi Arabia	5.29 (2.63, 10.11)	4.88 (2.99, 7.81)	0.05 (−0.18, 0.28)	3.17 (1.65, 6.36)	2.20 (1.48, 3.37)	−0.57 (−0.87, −0.27)	95.08 (47.90, 190.17)	63.06 (40.93, 97.71)	−0.93 (−1.16, −0.69)
South Africa	18.78 (11.95, 29.77)	47.54 (34.20, 69.67)	3.19 (2.75, 3.62)	11.07 (7.05, 17.89)	24.48 (18.15, 34.05)	2.64 (2.29, 2.99)	364.42 (235.38, 579.58)	834.46 (611.61, 1199.66)	2.89 (2.47, 3.31)
Turkey	5.65 (3.42, 8.85)	4.45 (3.11, 6.70)	−1.02 (−1.22, −0.81)	3.28 (1.98, 5.17)	1.82 (1.35, 2.53)	−2.00 (−2.18, −1.81)	103.92 (63.05, 162.21)	54.82 (39.83, 77.53)	−2.21 (−2.41, −2.02)
UK	36.30 (31.24, 42.42)	14.73 (12.20, 17.39)	−2.86 (−3.36, −2.35)	6.02 (5.56, 6.47)	2.60 (2.31, 2.92)	−2.51 (−2.82, −2.20)	214.30 (198.66, 231.12)	90.80 (81.07, 101.12)	−2.60 (−2.93, −2.27)
USA	26.46 (21.25, 33.45)	14.60 (11.64, 17.88)	−1.67 (−1.95, −1.39)	3.87 (3.42, 4.43)	2.65 (2.28, 2.99)	−0.99 (−1.10, −0.89)	142.37 (125.78, 162.68)	95.20 (82.19, 107.47)	−1.09 (−1.22, −0.96)

### 3.2. Comparison of age distribution of cervical cancer burden between China and G20 countries

**Figure 2.**
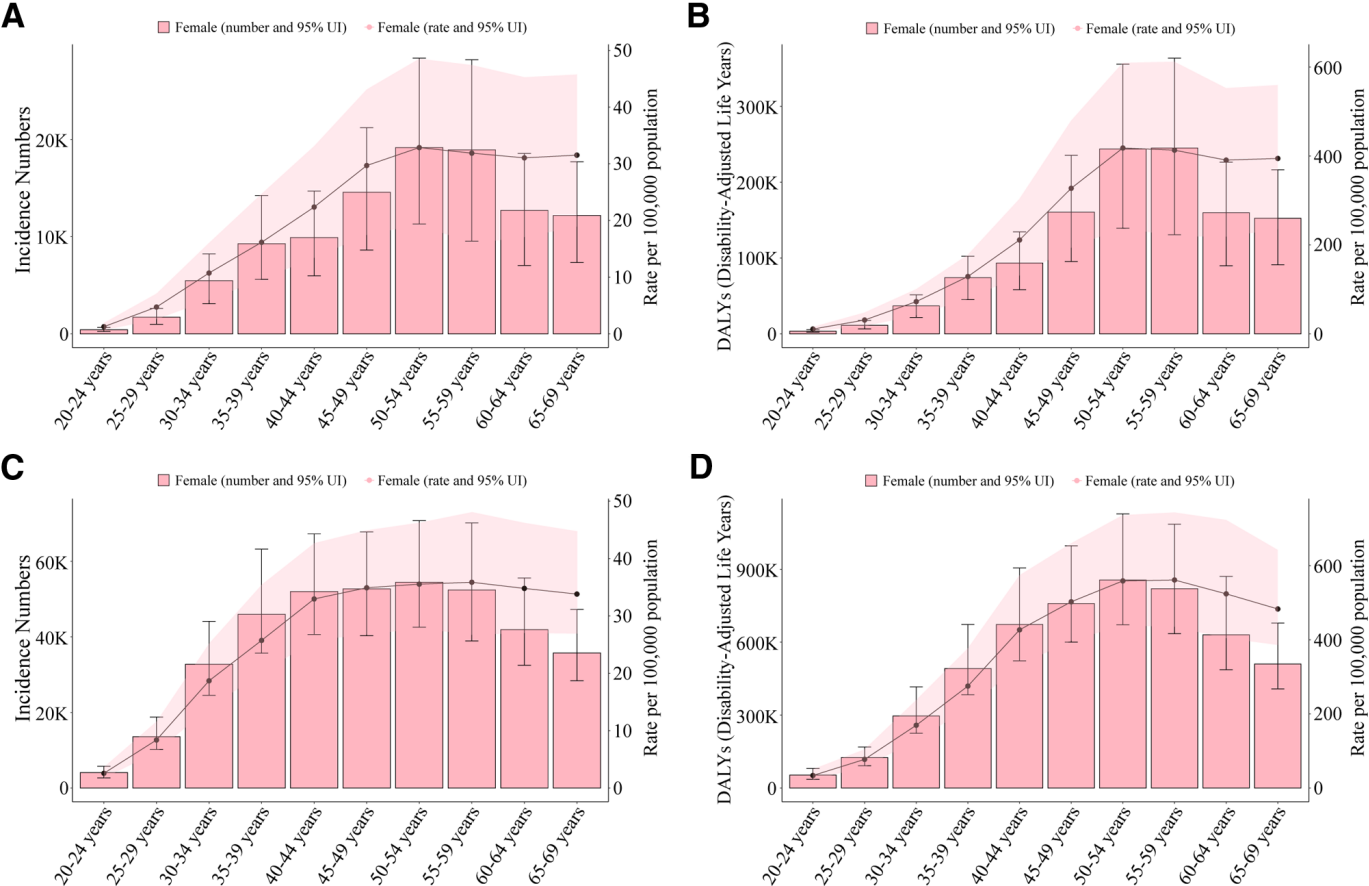
Age-specific distribution of cervical cancer incidence and disability-adjusted life years (DALYs) in China and G20 countries in 2023. Panels A/B: Cervical cancer incidence cases, DALYs, and DALYs rate in China; Panels C/D: Cervical cancer incidence cases, DALYs, and DALYs rate in G20 countries.

Age-specific analysis for 2023 revealed that the cervical cancer burden in both China and G20 countries peaked in the 50 to 54 age group. In China, the highest number of incident cases occurred among women aged 50 to 54, totaling 19,160 cases (95% UI: 11,306–28,355), with an incidence rate of 32.87 (95% UI: 19.39–48.64) per 100,000 (Fig. 2A). The DALYs burden peaked in the 55 to 59 age group at 245,078 (95% UI: 130,666–363,477) years, corresponding to a rate of 413.06 (95% UI: 220.23–612.61) per 100,000 (Fig. 2B). G20 countries exhibited a similar age distribution pattern, with the incidence peak likewise occurring in the 50 to 54 age group [54,431 cases (95% UI: 42,558–70,747); rate: 35.54 (95% UI: 27.79–46.20) per 100,000] (Fig. 2C). Notably, the DALYs rate for G20 countries in the same age group [558.84 (95% UI: 438.65–736.34) per 100,000] was markedly higher than the corresponding figure for China (Fig. 2D).

### 3.3. Decomposition analysis of cervical cancer burden in China and G20 countries

**Figure 3.**
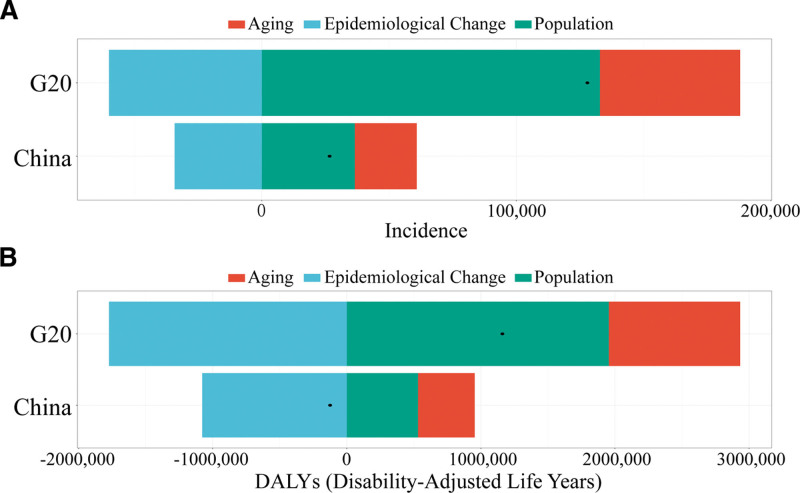
Decomposition analysis of changes in cervical cancer incidence and disability-adjusted life years (DALYs) in China and G20 countries from 1990 to 2023. Panel A: Decomposition of the overall change in cervical cancer incidence in G20 countries and China; Panel B: Decomposition of changes in DALYs attributable to cervical cancer over the same period.

To identify the drivers of changes in cervical cancer burden from 1990 to 2023, a demographic decomposition analysis was conducted. In China, the net increase in incident cases was 26,622, of which population aging contributed + 24,321 cases (+91.35%) and population growth contributed + 36,531 cases (+137.22%), while epidemiological change made a negative contribution (−34,230 cases, −128.58%), indicating a substantial decline in age-specific incidence risk across all age groups (Fig. 3A). With respect to DALYs, China achieved a net reduction of 124,436 years, as epidemiological improvements (−1078,043 years) successfully offset the combined effects of aging (+422,697 years) and population growth (+530,909 years) (Fig. 3B). In contrast, G20 countries experienced net increases in both incident cases (+127,907 cases) and DALYs (+1,160,254 person-years) (Fig. 3A,B). Population growth was the primary driver of the increase in case counts (103.85%), with epidemiological change only partially counterbalancing this effect (−46.94%). For DALYs, the upward pressure from population growth (168.42%) and aging (84.44%) exceeded the offsetting effect of epidemiological improvements (−152.87%).

### 3.4. Health frontier analysis and SDI correlations among G20 member states

**Figure 4.**
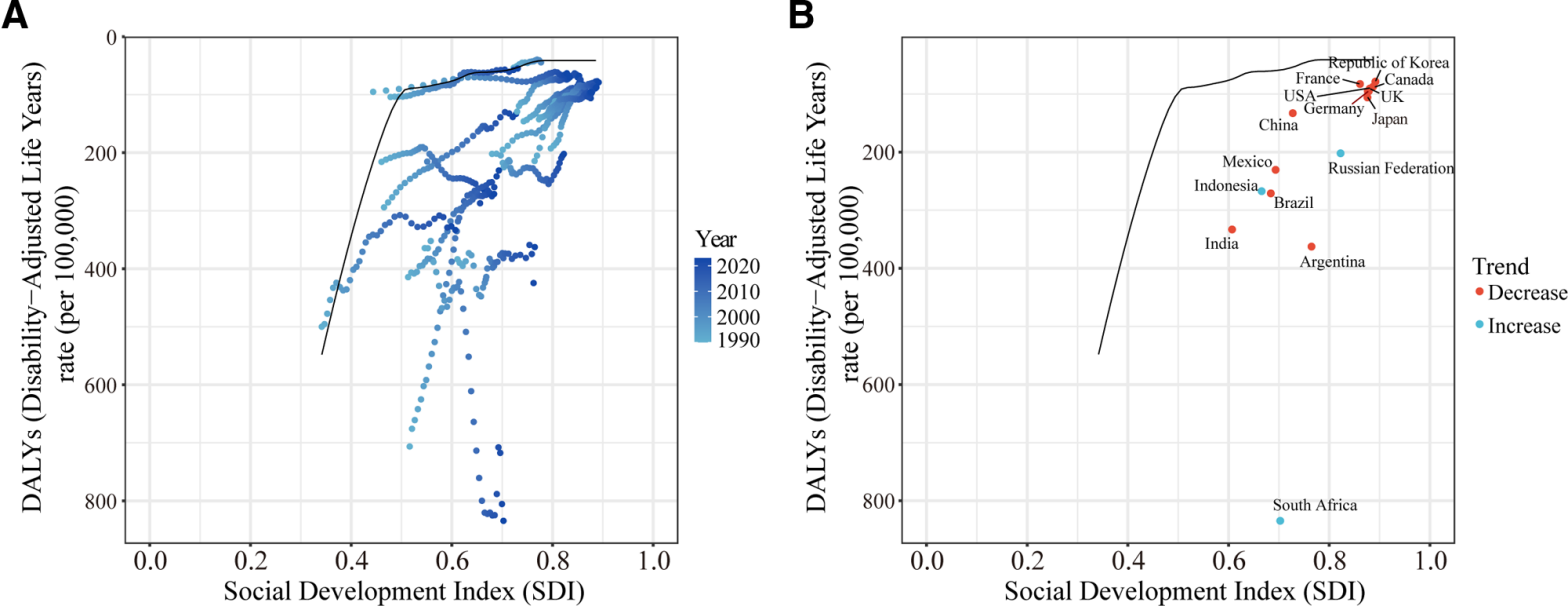
Frontier analysis based on the socio-demographic index (SDI) and age-standardized cervical cancer disability-adjusted life years (DALYs) rate in 2023. Panel A: Efficiency gaps between individual countries and the frontier level across each year from 1990 to 2023; Panel B: Actual gaps between individual countries and the frontier level in 2023 and 1990, respectively.

**Figure 5.**
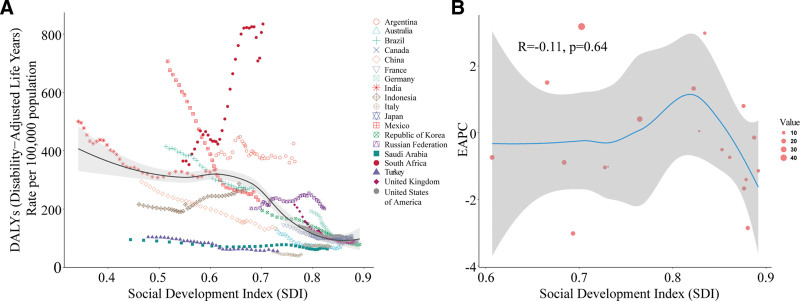
Relationship between age-standardized cervical cancer disability-adjusted life years (DALYs) rate and socio-demographic index (SDI) in G20 countries. Panel A: Temporal evolution of DALYs rates by SDI across countries from 1990 to 2023; Panel B: Correlation analysis between SDI and the estimated annual percentage change (EAPC) of DALYs rate.

Frontier analysis based on the SDI revealed substantial efficiency gaps between each country’s actual DALYs rate and the theoretical optimal performance across the study period (Fig. 4A). In 2023, South Africa had the largest efficiency gap at 775.67 (actual rate: 834.46 per 100,000 vs frontier value: 58.79 per 100,000), followed by Argentina (303.84) and India (274.38). China performed relatively well, with its actual DALYs rate (133.06 per 100,000) approaching the theoretical frontier value corresponding to its SDI level (0.72) (Fig. 4B). Spearman correlation analysis showed a significant negative correlation between SDI and cervical cancer DALYs rate among G20 countries from 1990 to 2023 (*r* = −0.5950, 95% CI: −0.6415–−0.5380, *P* < .001) (Fig. 5A). However, the correlation between SDI and the EAPC of incidence rate was weak and not statistically significant (*R* = −0.11, *P* = .64), suggesting that trends in incidence are influenced by multiple factors beyond socioeconomic development (Fig. 5B).

### 3.5. Projection analysis of cervical cancer mortality and death counts in China and G20 countries through 2035

**Figure 6.**
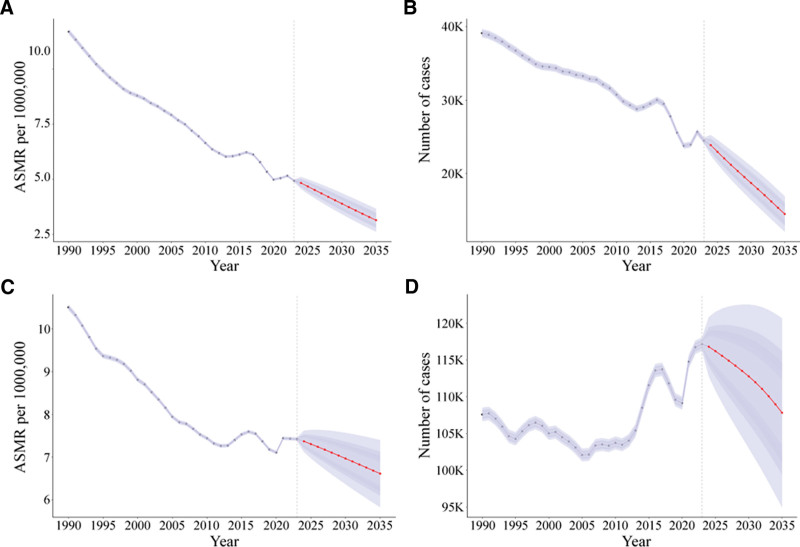
Temporal trends in age-standardized mortality rate (ASMR) and number of deaths from cervical cancer from 1990 to 2035. Panel A: ASMR in China; Panel B: Number of deaths in China; Panel C: ASMR in G20 countries; Panel D: Number of deaths in G20 countries.

A BAPC model was used to project mortality trends through 2035. In China, the ASMR is projected to decline from 4.19 (95% CI: 2.67–5.48) per 100,000 in 2023 to 3.14 (95% CI: 2.62–3.66) per 100,000 in 2035 (Fig. 6A). The number of deaths is expected to fall from 24,487 (95% CI: 24,081–24,893) in 2023 to 14,481 (95% CI: 12,072–16,891) in 2035, representing a reduction of 40.9% (Fig. 6B). For G20 countries, the model projects a transient increase in the ASMR between 2021 and 2024, with an anticipated peak of 7.37 (95% CI: 7.12–7.62) per 100,000 in 2024, followed by a gradual decline to 6.61 (95% CI: 5.83–7.40) per 100,000 by 2035 (Fig. 6C). The absolute number of deaths is projected to decrease from 117,148 (95% CI: 116,233–118,063) in 2023 to 107,831 (95% CI: 94,990–120,673) in 2035, a reduction of only 7.9% – far below the magnitude of decline projected for China (Fig. 6D).

## 4. Discussion

This study systematically compared trends in cervical cancer disease burden between China and G20 countries from 1990 to 2023 using the GBD 2023 database. The findings indicate that although cervical cancer burden declined in both China and G20 countries, China’s rate of decline was markedly faster than the G20 average, with a 52.4% reduction in ASMR – significantly greater than the 31.8% reduction observed in G20 countries – and an EAPC of −2.43 for the DALYs rate, also surpassing the G20 average. These trends are broadly consistent with findings from previous studies.^[17]^ Beyond this, the present study employed decomposition analysis to quantify the specific contributions of demographic factors and epidemiological changes to disease burden, offering a new perspective for understanding the mechanisms underlying shifts in disease burden. The rapid decline in cervical cancer disease burden in China may be attributable to multiple factors. First, the government-initiated free “two-cancer” (cervical and breast cancer) screening program in rural areas, launched in 2009, has expanded its coverage year by year, effectively improving the early detection rate of cervical cancer.^[6,7]^ Second, the pilot inclusion of HPV vaccination in the national immunization program in 2019 has gradually improved vaccine accessibility, particularly with the introduction and price reduction of domestically produced bivalent HPV vaccines, despite the current relatively low HPV vaccination coverage in China (approximately 9.5%).^[18,19]^ In addition, growing health awareness among Chinese women and improved accessibility of healthcare services have further supported cervical cancer prevention and control efforts. These findings suggest that China has achieved notable progress in cervical cancer control, particularly in the areas of screening system development, optimization of healthcare resource allocation, and implementation of public health interventions.

Through decomposition analysis, this study quantified the specific contributions of population growth, aging, and epidemiological changes to cervical cancer burden in China and G20 countries. The results indicate that epidemiological change was the primary driver of disease burden reduction. In China, epidemiological improvements (−128.58%) successfully offset the combined effects of population aging (+91.35%) and population growth (+137.22%), resulting in a net reduction of 124,436 DALYs years. This finding confirms the critical role of advances in medical technology, expansion of screening programs, and HPV vaccination in reducing cervical cancer burden.^[20]^ Notably, while epidemiological changes exerted a substantial negative contribution, population aging still exerted a positive (burden-increasing) effect. In China, the impact of population aging was particularly pronounced, consistent with the country’s rapid transition into an aging society. In contrast, although the relative magnitude of epidemiological improvement in G20 countries (−152.87%) was higher, it was insufficient to fully offset the burden-increasing effects of demographic factors, resulting in a net increase in DALYs. As the proportion of elderly individuals continues to rise, absolute disease burden may still increase even when age-standardized rates decline.

The results also revealed substantial heterogeneity in cervical cancer burden among G20 member states, reflecting differences in health systems, HPV vaccination coverage, and the reach of screening programs. South Africa recorded the highest incidence rate among G20 countries at 47.54 per 100,000, a finding closely linked to its high HIV prevalence. Evidence indicates that HIV-positive women face a 3.3 to 5.7 times higher risk of cervical cancer compared to HIV-negative women,^[21]^ and approximately 24 to 25% of cervical cancer cases in sub-Saharan Africa are attributable to HIV infection.^[22]^ The anomalous upward trend in cervical cancer mortality in Italy (EAPC: 2.06) may be associated with declining screening participation, an increasing immigrant population, and delays in HPV vaccination rollout.^[23]^ In stark contrast, Australia pioneered a national HPV vaccination program in 2007 and transitioned its screening strategy from cytology to primary HPV testing in 2017, and is projected to become the first country in the world to achieve cervical cancer elimination.^[24]^ This success demonstrates that combining high-coverage HPV vaccination with an efficient screening system is the key pathway to achieving cervical cancer elimination. Accordingly, each country should formulate targeted prevention and control strategies tailored to its own disease burden characteristics and healthcare resource conditions.

Frontier analysis results indicated that China’s actual DALYs rate (133.06 per 100,000) approached the theoretical optimum corresponding to its SDI level (0.72), suggesting that China has performed well in leveraging its cervical cancer prevention and control potential relative to its current level of socioeconomic development. However, the absence of a significant correlation between SDI and the EAPC of incidence rate (*R* = −0.11, *P* = .64) suggests that trends in cervical cancer incidence are shaped by multiple factors – including HPV vaccination policy, screening program coverage, and healthcare resource allocation – rather than being determined solely by socioeconomic development.^[25]^ Projections from the BAPC model indicate that cervical cancer deaths in China will decline by 40.9% from 2023 to 2035, compared to only 7.9% in G20 countries. Notably, the model projects a transient increase in mortality among G20 countries between 2021 and 2024, which may reflect the delayed impact of the COVID-19 pandemic on cervical cancer screening services – a phenomenon that has been reported in cancer screening data from multiple countries.^[26]^

The WHO’s Global Strategy to Accelerate the Elimination of Cervical Cancer, released in 2020, set the “90-70-90” targets: by 2030, to have 90% of girls fully vaccinated against HPV before age 15, 70% of women screened with a high-performance test between ages 35 and 45, and 90% of women with cervical disease receiving treatment.^[9]^ Benchmarked against these targets, China still has considerable room for improvement in both screening coverage and vaccination rates. Based on the above findings, we propose the following recommendations. First, HPV vaccination promotion should be accelerated, with particular emphasis on expanding the availability of domestically produced vaccines and implementing single-dose immunization strategies to reduce vaccination costs and improve accessibility. Second, the cervical cancer screening network should be further strengthened, with enhanced screening capacity at primary healthcare institutions to narrow the prevention and control gap between urban and rural areas and across regions. Third, screening coverage should be prioritized for women in the high-risk 50 to 54 age group, accompanied by strengthened health education and disease prevention outreach. Fourth, international cooperation and experience-sharing should be enhanced, drawing on the successful approaches of countries such as Australia to collectively advance the global agenda for cervical cancer elimination.

This study has several limitations. First, the GBD database draws on data from a variety of sources, and data quality and completeness may vary across countries. Second, decomposition analysis assumes that the driving factors are mutually independent; however, in practice, changes in population structure and epidemiological characteristics may interact with one another. Furthermore, the projection model was unable to fully account for the potential influence of policy factors such as future expansion of HPV vaccination and changes in screening strategies. Finally, the absence of province-level data precluded analysis of regional disparities within China, which represents an important direction for future research.

## 5. Conclusion

This study demonstrates that the cervical cancer disease burden in China declined significantly faster than the G20 average between 1990 and 2023, with epidemiological improvements effectively offsetting the adverse impacts of population aging and growth. Projections indicate that cervical cancer deaths in China will decrease by 40.9% from 2023 to 2035, reflecting an optimistic prevention outlook. Nevertheless, substantial room remains for improving HPV vaccination coverage and screening accessibility, with urban-rural and regional disparities still to be addressed. Sustained efforts to expand HPV vaccination programs and strengthen cervical cancer screening systems will be essential to achieving the WHO’s strategic goal of cervical cancer elimination.

## Acknowledgments

The authors sincerely thank the Global Burden of Disease Study 2023 collaborators and the Institute for Health Metrics and Evaluation (IHME) at the University of Washington for making the GBD 2023 database publicly available. The findings and conclusions in this paper are those of the authors and do not necessarily represent the views of the IHME or its funders.

## Author contributions

**Conceptualization:** Jing Li.

**Data curation:** Jing Li, Ying Wang.

**Formal analysis:** Ying Wang, Zhi Xu.

**Investigation:** Ying Wang, Zhi Xu.

**Methodology:** Zhi Xu, Sisi Ao.

**Software:** Ying Wang, Zhi Xu.

**Supervision:** Sisi Ao.

**Writing – original draft:** Jing Li, Zhi Xu.

**Writing – review & editing:** Sisi Ao.
